# The interplay of chemical structure, physical properties, and structural design as a tool to modulate the properties of melanins within mesopores

**DOI:** 10.1038/s41598-022-14347-y

**Published:** 2022-07-06

**Authors:** Alessandro Pira, Alberto Amatucci, Claudio Melis, Alessandro Pezzella, Paola Manini, Marco d’Ischia, Guido Mula

**Affiliations:** 1 PoroSiLab, Dipartimento di Fisica, Università degli Studi di Cagliari, Cittadella Universitaria di Monserrato, S.P. 8 km 0.700, 09042 Monserrato (Ca), Italy; 2 Dipartimento di Fisica, Università degli Studi di Cagliari, Cittadella Universitaria di Monserrato, S.P. 8 km 0.700, 09042 Monserrato (Ca), Italy; 3 Dipartimento di Fisica “Ettore Pancini”, Università di Napoli “Federico II”, Complesso Universitario Monte S. Angelo, Via Cintia 21, 80126 Napoli (Na), Italy; 4 Dipartimento di Scienze Chimiche, Università di Napoli “Federico II”, Complesso Universitario Monte S. Angelo, Via Cintia 21, 80126 Napoli (Na), Italy

**Keywords:** Electrochemistry, Porous materials, Materials for devices, Nanoscale materials, Theory and computation

## Abstract

The design of modern devices that can fulfil the requirements for sustainability and renewable energy applications calls for both new materials and a better understanding of the mixing of existing materials. Among those, surely organic–inorganic hybrids are gaining increasing attention due to the wide possibility to tailor their properties by accurate structural design and materials choice. In this work, we’ll describe the tight interplay between porous Si and two melanic polymers permeating the pores. Melanins are a class of biopolymers, known to cause pigmentation in many living species, that shows very interesting potential applications in a wide variety of fields. Given the complexity of the polymerization process beyond the formation and structure, the full understanding of the melanins' properties remains a challenging task. In this study, the use of a melanin/porous Si hybrid as a tool to characterize the polymer’s properties within mesopores gives new insights into the conduction mechanisms of melanins. We demonstrate the dramatic effect induced on these mechanisms in a confined environment by the presence of a thick interface. In previous studies, we already showed that the interactions at the interface between porous Si and eumelanin play a key role in determining the final properties of composite materials. Here, thanks to a careful monitoring of the photoconductivity properties of porous Si filled with melanins obtained by ammonia-induced solid-state polymerization (AISSP) of 5,6-dihydroxyindole (DHI) or 1,8-dihydroxynaphthalene (DHN), we investigate the effect of wet, dry, and vacuum cycles of storage from the freshly prepared samples to months-old samples. A computational study on the mobility of water molecules within a melanin polymer is also presented to complete the understanding of the experimental data. Our results demonstrate that: (a) the hydration-dependent behavior of melanins is recovered in large pores (≈ 60 nm diameter) while is almost absent in thinner pores (≈ 20 nm diameter); (b) DHN-melanin materials can generate higher photocurrents and proved to be stable for several weeks and more sensitive to the wet/dry variations.

## Introduction

Porous materials are becoming increasingly relevant thanks to their low fabrication costs and their large surface-to-volume ratio. Their application range spans from biosensors^1^ to energy harvesting and production^2–4^. Excitonic solar cells are one example of their application and provide highly attractive and promising solutions for solar energy conversion^5^. At present, the development of robust and highly efficient photovoltaic devices using Si-based materials, in particular organic–inorganic hybrids, is however hampered by the trap-limited diffusion processes that mediate electron transport hampering the energy production process, especially at the longer visible-light wavelengths^6,7^. Organic materials offer significant assets in terms of costs, flexibility and processability^8,9^, and environmental impact^10^, but, compared to inorganic materials, they usually show lower light absorption coefficients and worse electronic performances^11^. Organic–inorganic hybrid devices offer therefore an interesting mix of the properties of both components, since they combine the low fabrication costs and versatility of the organics with the usually higher efficiency of the inorganics^12,13^. In this frame, the possibility of interfacing an inorganic matrix with an organic material, such as a conductive polymer in organic/inorganic nanocomposites, has greatly expanded the range of application of porous structures as energy materials^14–17^.

An attractive inorganic substrate for the development of innovative hybrid materials is porous silicon (PSi). PSi has been studied for a very wide range of applications, going from biomedical devices^18^ to biosensors^19–22^, to drug delivery systems^23–28^ and to optoelectronics^29,30^. In the field of energy applications, PSi has been used as an antireflection coating for crystalline Si^31–34^ and the realization of plasmonic layers for solar cells and sensing has been explored^35,36^. However, at present there are very few studies focused on its photovoltaic properties^37–40^, even if porous Si structures allow the fabrication of bulk heterojunctions, that is structures where the large surface-to-volume ratio may significantly enhance the photogeneration process^41,42^.

Different strategies are used in order to functionalize the different three-dimensional shapes of PSi and enhance its photoconductivity. An interesting example of application of porous silicon structures for photovoltaics is using poly(3,4-ethylenedioxythiophene) polystyrene sulfonate (PEDOT:PSS) in a Si nanowire/PEDOT:PSS heterojunction as low-cost solar cell^43^. The control of the SiO_x_ passivation layer is further tool to improve the interaction between silicon and PEDOT:PSS in the blend, thus resulting in improved photoconducting performances^44^. Polyaniline represents another interesting polymer interfaced with PSi^45–47^. Spin coating and electropolymerization of polyaniline (PANI) onto PSi^47,48^ showed an enhancement of electrical properties and photoconductance, although some biocompatibility problems exist for PANI^49,50^.

Synthetic eumelanins mimicking the natural black pigment of human skin, hair and eyes have also recently been the focus of interest as conductive soft biocompatible materials because of their unique optoelectronic properties^51^. Starting from this evidence, a promising organic–inorganic hybrid material, consisting of a bulk heterojunction made of porous silicon (PSi) and eumelanin, formed in situ by solid state polymerization of 5,6-dihydroxyindole (DHI)^38,40,52^, was tested for photovoltaic applications. The relatively intense and broad absorption spectrum of eumelanin was found to enhance the light absorption capabilities of the empty porous silicon matrix in the red and infrared spectral range, with a particularly marked increase of the photocarrier collection efficiency at longer wavelengths^38^. The polymerization of DHI in n^+^-doped porous silicon (n-PSi) was produced in situ by ammonia-induced solid-state polymerization (AISSP)^53^ after the pores were filled with DHI^38,52^. With this hybrid, a relatively intense photocurrent density up to 3.8 mA/cm^2^ was measured upon irradiation with visible light.

A persistent drawback for the photocurrent generation with PSi:eumelanin structures was found to be the short lifetime of the photocurrent generation process^40,54^. Despite the promising features of the PSi–eumelanin interface produced by the AISSP protocol, several issues remain to be settled, including the low efficiency, the marked instability, and the limited reproducibility. To better understand the complexity of the hybrid eumelanin/Si interface and to design efficient and stable PSi–eumelanin-based devices, we studied the molecular mechanisms of eumelanin buildup and adhesion on the inorganic substrate^40^. A main goal of this effort was the control of the parameters that govern the adhesion, interaction and electron–ion transfer at the organic/inorganic interface, to enable the optimization of the photocurrent generation. As observed in previous work, the pore diameter in the PSi:melanin structures plays a very important role in final properties of hybrid^54^, leading to an improved stability of the hybrid samples photocurrent lifetime. This improved stabilization has been attributed, using computational modelling, to the presence of a denser polymer for a thickness up to 5 nm from the Si/polymer interface, whose relevance for the polymers properties decreases as the pore diameter increases.

For this reason, in this work we first decided to experimentally investigate this aspect by studying the behavior of macroporous PSi samples (pore diameter ≈ 60 nm) impregnated with melanins.

**Figure 1:**
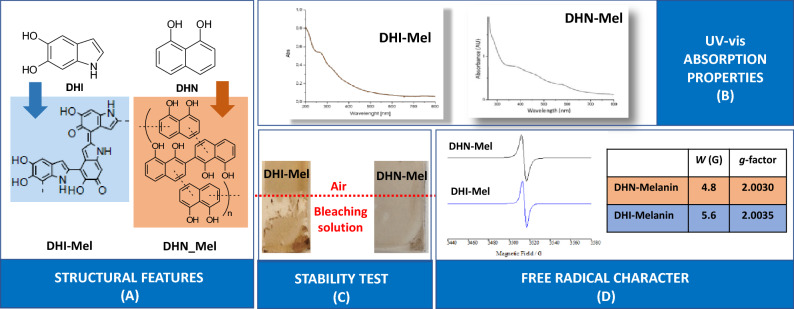
Pictorial view of the main properties of the melanin polymers obtained from DHI and DHN. In detail, (**A**) shows the main structural features, (**B**) shows the UV–visible absorption profiles, (**C**) shows the different robustness and (**D**) shows the EPR spectra and main data^58–62^.

A second set of measurements aimed at testing the performances of a different type of hybrid, the PSi:allomelanin hybrid, obtained by carrying out the AISSP protocol on 1,8-dihydroxynaphthalene (DHN). DHN is a metabolite and the monomer precursor of the nitrogen-free allomelanin pigment found in some fungi (i.e. Ascomyces)^55^ that recently has gained particular interest not only for its biological function but also for a series of chemical and physical properties allowing for its application in different sectors^56,57^. As summarized in Fig. 1, the choice of these two synthetic melanin polymers stems from the structural diversity of the monomer precursors, DHI and DHN, responsible for some different properties exhibited by the polymers.

Despite a series of common properties typical of the melanin pigments^58,59^, including the broadband absorption throughout the entire UV–visible spectrum, the intrinsic free radical character, and the formation of quasi-spherical supramolecular aggregates, the DHI and DHN melanin polymers exhibit a different distribution of the unpaired spins along the polymer backbone. This accounts for a more pronounced C-radical character in the case of DHN-melanin and O-radical character in the case of DHI-melanin. Finally, a different chemical stability has been observed pointing out the higher robustness of DHN-melanin^60–62^.

The results presented in this study will show how the larger pore diameter can dramatically modify the stability and performances of the PSi:melanin hybrid, improving the stability from a couple of hours to several months and increasing by at least one order of magnitude the photocurrent values.

We will also present a comparative investigation of the performances of the PSi:eumelanin and PSi:allomelanin materials, pointing out for the first time the higher stability and photocurrent generation of the latter hybrid.

## Experimental and simulation details

### Porous Si layers

Prior to PSi fabrication, the Si surfaces were prepared using the Electrochemical NanoLithography (ENL) process to control the density, distribution, and size homogeneity of the PSi pores. ENL is a patented^63^ three-steps nanolithography process, developed by some of the authors^64^, aimed at the control of the pore’s density, size homogeneity and opening size distribution regularity. The ENL process is characterized by the formation of a porous double layer that is etched away after formation using aqueous 0.5 M NaOH cold solution to leave a nanostructured surface whose holes serve as seeds for the pore nucleation in the third layer. After the ENL process, that is having obtained a top Si surface with controlled indentations, the choices of HF concentration, current intensity and etching time give control over the pore diameter and shape for the PSi layer to be impregnated.

**Figure 2:**
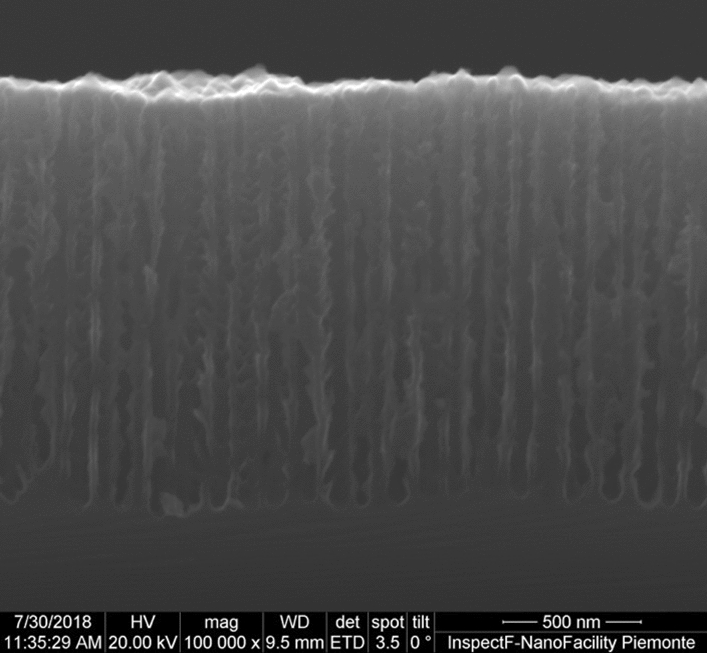
SEM image of the cross section of a typical sample, where the pore shape and size are visible.

PSi layers have been fabricated by electrochemical etching in the dark of n^+^ Si wafers (ρ = 15–18 mΩ/cm, from Sil’tronix, France). The etching was performed using several HF concentrations in a HF/H_2_O/EtOH solution for the ENL process^64^. The electrochemical fabrication process for the final layer was performed using a solution of HF:H_2_O:Ethanol in 21:21:58 proportions, respectively, and a current density of 900 mA/cm^2^^64^. The Scanning Electron Microscopy (SEM) image of the cross section of a sample fabricated using those parameters is shown in Fig. 2. HF is a highly corrosive acid that may be lethal if manipulated without the necessary personal protection equipment and the correct chemical laboratory tools. For reference see, for instance, the University of North Carolina web pages dedicated to the HF safety procedures^65^.

The PSi pores were treated after formation using a diluted NaOH aqueous solution (0.1 M) to enlarge the pores to widen the pore diameter and smooth the pore walls. The pores enlarging process durations were 0 (untreated), 30 and 60 s.

### DHI and DHN fabrication

DHN has been purchased from Sigma Aldrich and used as is. DHI has been synthesized according to a procedure reported in the literature^66^.

### Porous Silicon/Melanin Hybrids

Porous silicon/melanin hybrids have been prepared by using the AISSP technique; in brief, first the impregnation of the PSi pores was obtained by spin coating using a 7 µL drop of an ethanolic solutions of DHI or DHN (the monomer concentration was 2.5 mg/250 µL), then the PSi was exposed to air saturated with ammonia vapors to promote the in situ formation of the melanin polymer^53^. The details of the impregnation process have been described elsewhere^40,54^, and the AISSP duration in these experiments was 600 s.

### Photocurrent measuring procedure

Semitransparent gold contacts, in order to make the photocurrent measurements, were deposited by sputtering onto the surface of all samples using an Emitech K450 sputter coater. All samples have been placed in the sputter chamber and treated for 6 min in Ar atmosphere using a sputtering current of 25 mA.

**Figure 3:**
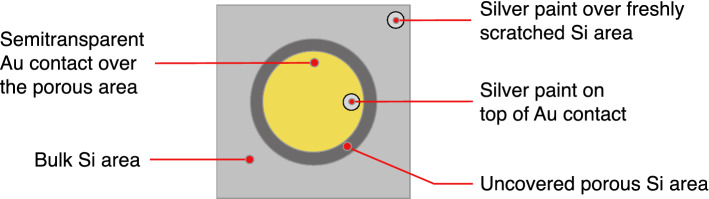
Schematic of a PSi sample ready for the photocurrent measurements. The various elements are indicated.

The gold deposition area diameter is 2 mm smaller (7 mm) than the PSi area diameter (9 mm) to ensure that no contact between the semitransparent gold layer and the bulk Si region is made. The surface of the semitransparent gold contact is 0.38 cm^2^. The percolation threshold of the gold film was taken as the reference for the amount of deposited gold. Following the sputtering process, a first silver paint drop has been placed onto the gold layer and a second one has been put on a freshly scratched area of the bulk Si substrate to ensure a contact to the bottom of the PSi layer^67^. A scheme of a sample ready for the photocurrent measurements is shown in Fig. 3. The samples photoconductivity was tested in a PM8 Analytical probe using a tungsten halogen lamp and a Keithley 2640 multimeter. No external voltage was applied during the photocurrent measurements. The current values were recorded after stabilization of the photocurrent values (two minutes).

### Storage treatment

Impregnated samples have been stored in alternated wet and dry environments. Samples stored in “dry” conditions were kept in a glass sealed container with standard desiccant. The box was equipped with a humidity detector in order to keep the atmospheric humidity level below 5%. Samples stored in “wet” conditions were covered with 250 µL of deionized water. The presence of water above the samples’ surface was regularly monitored. Before measuring, the samples kept in wet environment were dried using compressed air at 2 bars, in order to both disperse the water and avoid that Ag particles from the silver paint contact could diffuse onto the sample’s surface. This treatment prevented that the possible presence of dispersed Ag particles could alter the measured photocurrent output. The initial storage duration explored within this work ranged from 1 to 3 days. We observed no additional effects for longer initial storage durations.

### Synoptic view of the experimental steps

**Figure 4:**
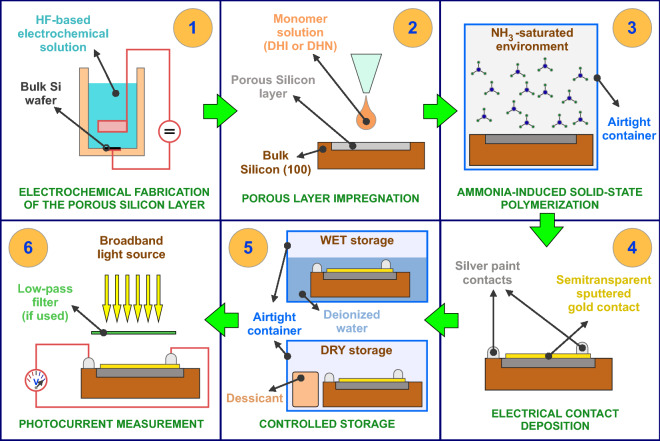
Schematic of the experimental steps of this study. (1) Fabrication of the porous layer, (2) impregnation of the porous layer using an alcoholic monomer solution, (3) polymerization of the monomers by keeping the samples in an ammonia-saturated environment, (4) deposition of the electrical contact, (5) wet or dry storage of the samples, (6) measure of the photocurrent values.

The various steps of the experimental procedure followed within this study are summarized in Fig. 4. The details of each step have been previously described in this section.

### Computational modelling

The hybrid eumelanin/silicon interfaces have been modelled by means of model potential molecular dynamics using the same procedure described by Antidormi et al.^54^. In detail, the silicon–silicon interactions are described by the Tersoff model potential^68^ and all the interactions between the eumelanin protomolecules, were modelled using the GAFF force field^69,70^. The atomic partial charges have been estimated using the restrained electrostatic potential (RESP) method^71^ using an HF/6-31G* QM calculation as implemented in the Gaussian package^72^. The eumelanin–silicon interaction was modelled by a combination of nonbonding and bonding terms: the covalent silicon–oxygen interaction is modelled via a reactive Tersoff potential^54^ and all the remaining nonbonding interactions are described by Lennard–Jones and Coulomb potentials^73^.

All the molecular dynamics simulations have been performed using the LAMMPS^74^ package by means of the velocity-Verlet algorithm with a time step of 0.5 fs to solve the equations of motion. A particle–particle mesh solver is used for describing the electrostatic interactions, and a cutoff 0.1 nm have been used for the van der Waals interactions. The temperature was controlled using the Nose’-Hoover thermostat with relaxation time equal to 50 fs.

The eumelanin/silicon samples contained 5400 eumelanin protomolecules and a silicon (100) 25 × 25 slab having in total 311 600 atoms. As previously described in detail^54^, the sample generation was obtained using a multistep procedure in which the eumelanin protomolecules were initially randomly placed in a region above the substrate and then deposited onto the silicon substrate for 10^6^ time-steps. A further 10^6^ time-steps at constant-temperature T = 300 K were performed during which the density profile was sampled.

## Results and discussion

Eumelanin polymers exhibit a multi-functional and functionalizable heteroaromatic platform that combines broadband visible light absorption, a water-dependent hybrid ionic-electronic semiconductor behavior, a distinct free radical character and a peculiar redox reactivity based on catechol/semiquinone/quinone conversion equilibria in the solid state and on the aggregate surface^75–79^. In recent experiments, however, the reported strong water-dependent conductivity^75,76^ was feebly visible when the DHI-based melanins were kept within tiny pores (10-15 nm diameters) while was more present in the larger pores, as reported by Antidormi et al.^54,80^.

### Effect of the average pore diameter

On these bases, the first step of this study was to demonstrate the correlation between large (> 50 nm) pore diameter and the overall bulk-like behavior of the melanins. In particular, we aimed at measuring the dependence of the photocurrent intensity in impregnated PSi samples on dry/wet storage environments for larger pores. In the hypothesis of Antidormi et al.^54,80^, the presence of a 5 nm-thick interface indicates that the larger pores should allow the polymer to recover its bulk structure and, therefore, to show the same behavior both if infiltrated within pores or as a film on a planar surface.

**Figure 5:**
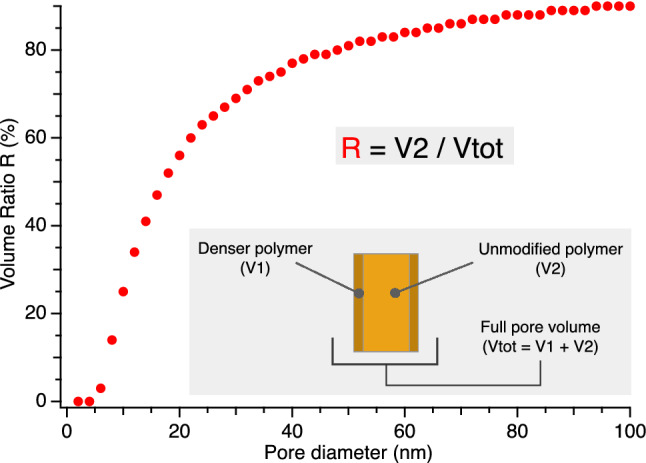
Evolution of the ratio R between the unmodified polymer volume V2 to the full pore volume Vtot as a function of the pore diameter. The symbols are defined in the figure inset. Larger pore diameters lead to higher R, with the highest sensitivity in the 5–30 nm diameter range.

The expected dependence of photocurrent from pores size comes from the fact that in a filled pore of total volume Vtot, the repartition of the space occupied by the denser polymer (V1) and the bulk-like polymer (V2) vary when going from tiny to large pores. For equal pore lengths, a pore with 60 nm diameter has an inner volume Vtot almost 10 times larger than that of a pore with 10 nm diameter. Let us consider a 5 nm-thick polymer/silicon interface^40,54^, that is the thickness of the denser polymer close to the Si surface. In a 60 nm diameter filled pore, about 70% of Vtot is occupied by the “bulk-like” polymer (V2). However, in a pore with 20 (15) nm diameter, the unaffected polymer occupies only 25% (11%) of Vtot. In Fig. 5 we show the evolution of the volume ratio, R = V2/Vtot, as a function of the pore diameter.

This large R difference between tiny and large pores is expected to produce a relevant modification on the overall infiltrated polymer properties and, therefore, on the hybrid material properties, as our results will demonstrate. Since the conductivity properties of the melanin within the pores will affects the overall conductive behavior of the PSi/melanin samples and therefore of the value of the measured photocurrent intensity, we know that variations in a given sample’s photocurrent measurements as a function of the wetting parameters are directly related to the variations of the melanin conductivity. Please note that in the following data analysis, we will use the initial “W” and “D” letters to indicate an initial wet or dry environment, respectively, followed by DHI or DHN to indicate what monomer was used to obtain the melanin. The number after D or W indicates the duration (in days) of the initial wet or dry storage environment.

### Effect of the wet and dry storage on DHI-based melanin

**Figure 6:**
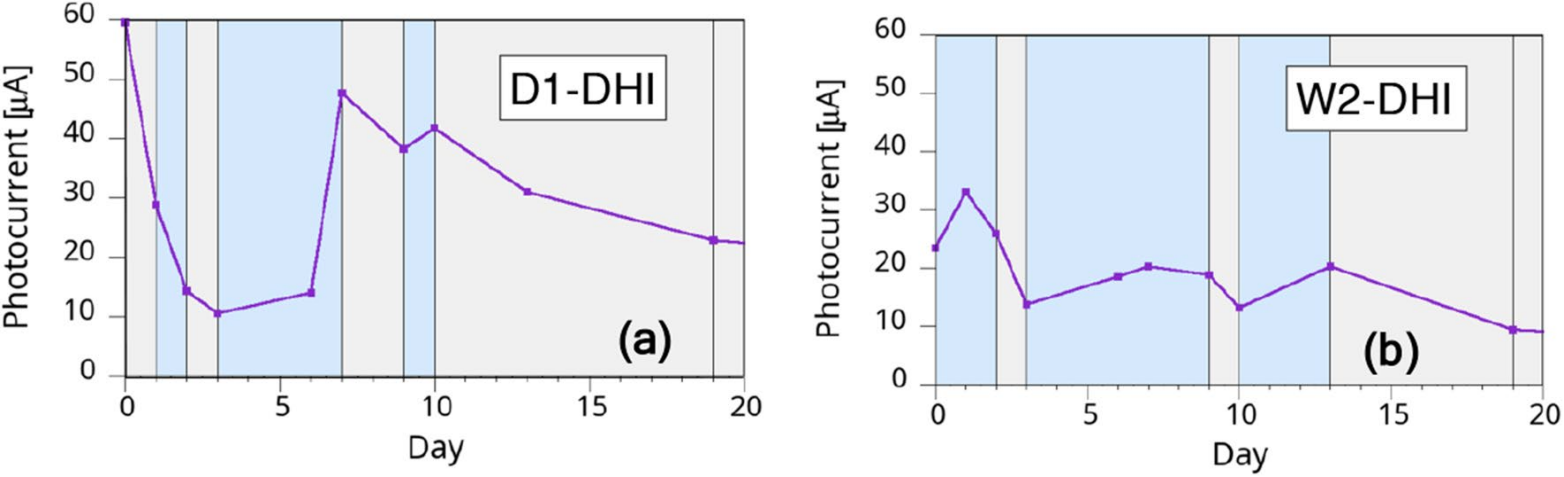
Behavior of PSi:melanin samples when stored in wet (light blue areas) and dry (grey areas) environment for (**a**) D1-DHI sample and (**b**) W2-DHI sample in a timespan of three weeks after formation.

To test the humidity dependence of the samples' properties, as a first attempt we fabricated a set of PSi samples with DHI-based melanin and divided the samples in two sets: after formation, part of the samples was initially kept in dry conditions (D-DHI) and the rest in wet conditions (W-DHI), alternating the storage environment after a few days. If the humidity degree affects the photocurrent measurements, this will indicate that the infiltrated polymer changes its conducting properties as it does for thick layers. We therefore expect that W-DHI samples will lower their photocurrent intensity after being put into dry conditions, and vice versa for the D-DHI samples. To ensure the correct interpretation of our results, the dark current value was routinely monitored in each measurement and was measured in all cases as being between 3 and 5 orders of magnitude smaller than the white-light photocurrent values. The dependence on the hydration level of the dark photocurrent, if present, was below our detection limit. The effect of hydration on DHI-impregnated samples is shown in Fig. 6, for one sample initially kept in a dry environment (left) and one initially kept in a wet environment. The white-light photocurrent intensity from the samples has been reported as a function of the time (days) since the fabrication. It is worth noting that, for the measurements to be reliable, we established a two minutes stabilization delay before recording the measurement. The waiting time has been kept identical for all samples in this work. We also routinely measured the spectral dependence of the photocurrent using low pass filters^38^ as a preliminary check for the reliability of the experiments (data not shown).

The results in Fig. 6, while still not exhibiting dramatic changes, show that the observed behavior is coherent with the hypothesis for the wet/dry dependence of the photocurrent intensity but also shows increased stability in time, indicating therefore that the polymer has at least partially recovered its bulk properties. The observed behavior for samples with thinner pores was in fact quite different: not only the wet/dry cycles didn’t show marked differences between the two storage conditions, but these small differences disappeared quickly after a couple of cycles^52^.

An interesting aspect of this first set of measurements was the fact that we observed that the conductivity modification from wet to dry environments and vice versa stabilized after 2–3 days from the environment change, indicating a slow dynamic of the process.

### Comparison of DHI and DHN melanins

To gain additional insight on the PSi:melanin hybrid, we compared the performances of nominally identical samples impregnated with DHI or with DHN.

As shown in Fig. 1, DHI and DHN exhibits quite different structural features that confer to the corresponding melanin polymers different characteristics. While both DHI- and DHN-melanin have similar optical and morphological properties^60^, DHN leads to melanin polymers characterized by a more intense paramagnetic character (evidence by Electron Paramagnetic Resonance, EPR) and a higher stability (chemical degradation tests) when compared to DHI-melanin^61^.

**Figure 7:**
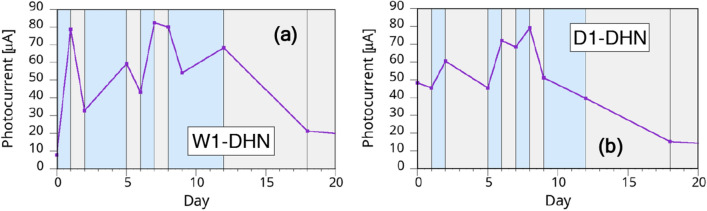
Behavior of PSi samples with DHN-based melanin: (**a**) W1-DHN sample and (**b**) D1-DHN sample. The grey and light blue areas indicate dry and wet storage environment, respectively.

In Fig. 7 we show the results for typical W1-DHN and D1-DHN samples. While the maximum photocurrent remains about the same as for W2-DHI and D1-DHI samples, the variations as a function of the wetting are more marked. The second relevant difference with DHI-based melanins is the improved reproducibility of the DHN samples behavior from sample to sample. As shown in the figure, measurements made after an interval of less than a couple of days from the environment change, while in general following the wet/dry path, may occasionally suffer from measurement noise since the behavior is still not stabilized.

### Initial samples conditioning and lifetime increase

**Figure 8:**
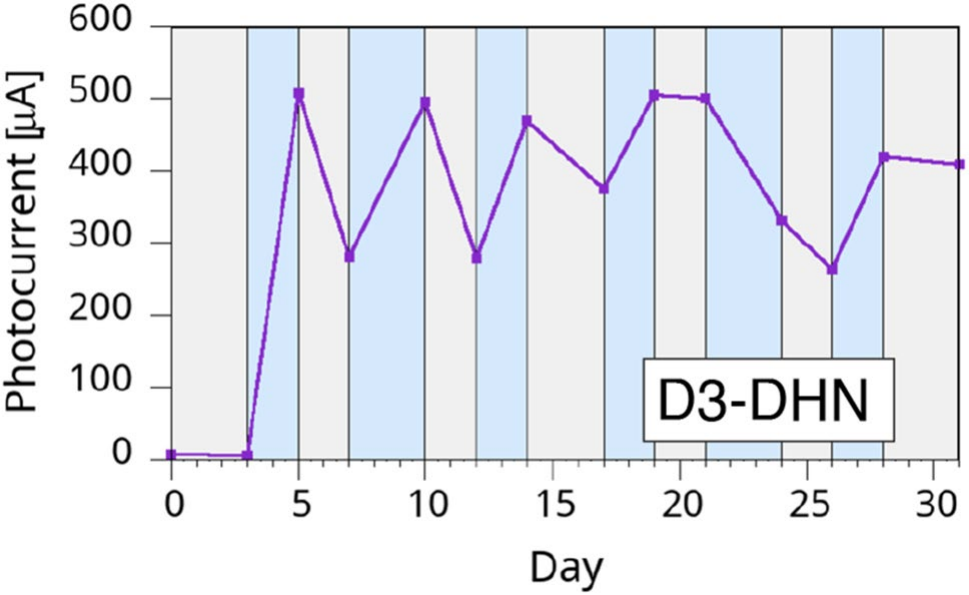
Temporal evolution of the photocurrent generation in a D3-DHN sample. The grey and light blue areas indicate dry and wet storage environment, respectively.

To better understand the reasons for the observed fluctuations from sample to sample, we explored the effect of the samples’ treatment in the first days after fabrication, focusing our study on the use of the stabler DHN-based samples. In particular, after the initial “as-prepared” measurement (that is after the contact deposition) we stored the samples in three different environments: dry, wet and low vacuum. All samples stored in vacuum showed little or no photocurrent generation behavior and no dependence on the following wet or dry environment, so we dropped this initial treatment early on the tests to focus our attention on the wet and dry environments. We chose to keep the initial treatment for 2 to 3 days, since the slow dynamics observed for the stabilization of the photoconducting behavior strongly suggests that a shorter delay does not allow a full stabilization of the effect. In our experiments, samples treated in wet and dry environment showed significant differences. W3-DHN samples showed relevant fluctuations in the photocurrent values and in their overall behavior, while D3-DHN samples showed a significantly more marked sensitivity to the environment and stabler results. The results for a typical D3-DHN sample are shown in Fig. 8.

The first remarkable information from the results in Fig. 8 is that when a D3-DHN sample is put in a wet environment the measured photocurrent raises up to about two orders of magnitude with respect to the initial measured value. Second, the samples sensitivity to the wet/dry variations are significantly higher than in previous samples. Furthermore, D3 samples show another remarkable improvement with respect to the previous sets: the overall photocurrent values were stable for several weeks, remaining on values 10 to 100 times larger than the initial values, depending on the samples. This is by itself a remarkable difference between the two melanins, since in our past experiments with DHI^40,54,80^, while 60 nm-diameter pores gave better stability than for 15 nm-diameter pores, nevertheless the samples showed that a month after fabrication the photocurrents were at most about 20% of the initial value.

A possible interpretation of the DHN sample behavior may be offered by the well-established high stability exhibited by the DHN-melanin (see Fig. 1)^61^, that probably is less prone to undergo redox and other degradation processes that may occur during the course of the many wet/dry cycles.

### Long term temporal stability

**Figure 9:**
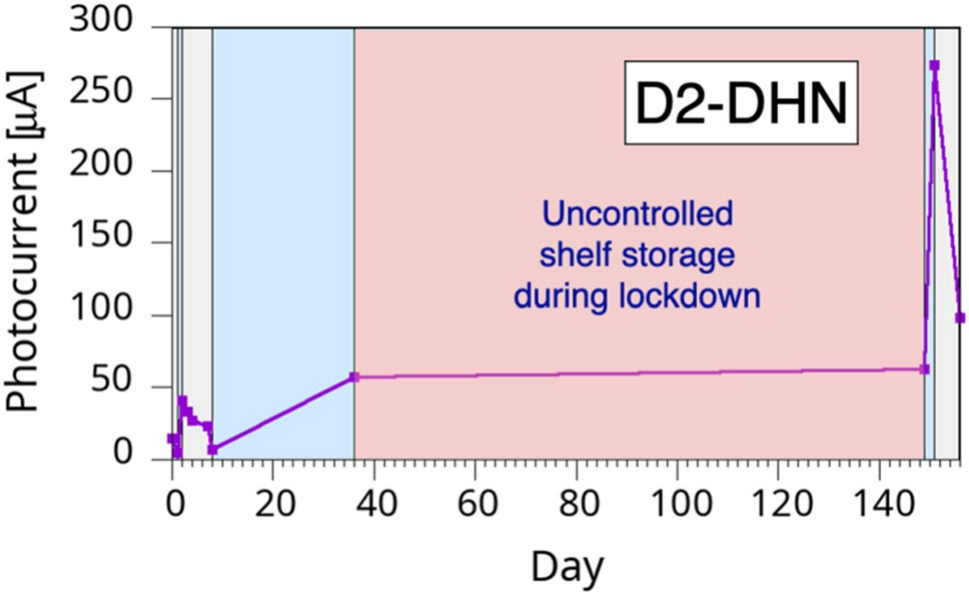
Temporal evolution of a D2-DHN sample photocurrent. The grey and light blue areas indicate dry and wet storage environment, respectively. The light red area indicates the temporal interval corresponding to the Covid-19 lockdown restrictions to laboratory access.

**Figure 10:**
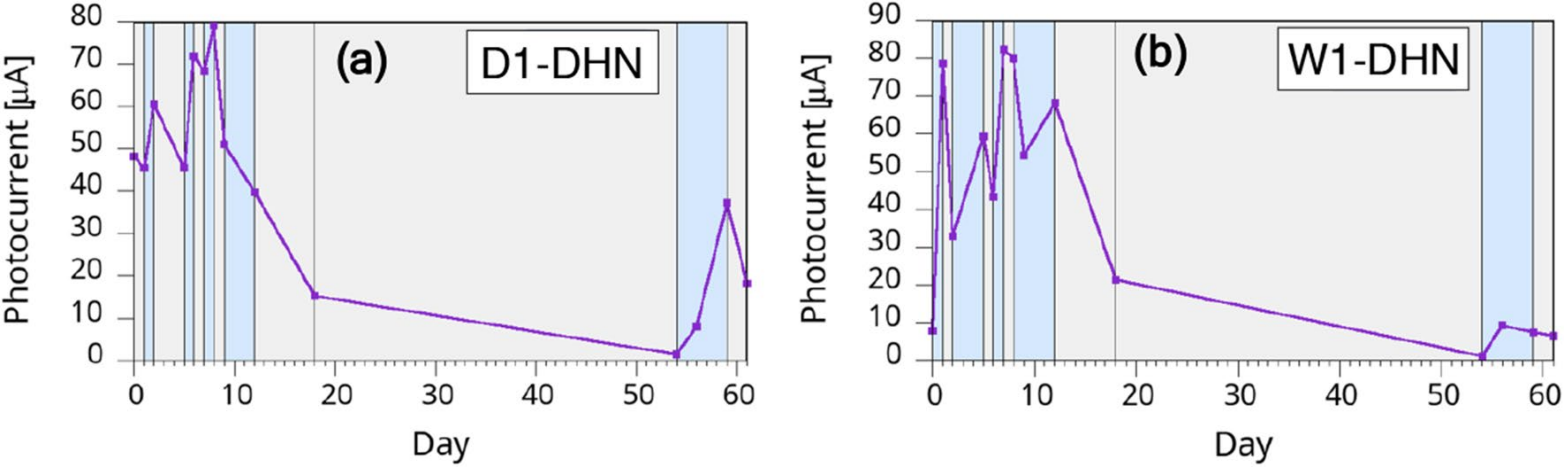
Long-term evolution of the (**a**) D1-DHN and (**b**) W1-DHN samples shown in Fig. 6. The grey and light blue areas indicate dry and wet storage environment, respectively.

We tested the temporal stability on several samples from all categories cited in this study for several months. A number of samples were measured after two months of uncontrolled shelf life, when we had to interrupt the measurements for the Covid-19 related restrictions in Italy. In Fig. 9 we show how these samples maintained an excellent behavior even after the poorly controlled shelf life (evidenced by the light-red shaded area), demonstrating then how the larger pores and the initial dry treatment greatly improved the melanin/PSi performances and lifetime. For comparison, in Fig. 10 we show the long-term (two months) photogeneration behavior of DHN samples whose initial treatments in dry environment lasted for only one day. The samples are the same two shown in Fig. 6. While the better ability to generate photocurrent after two months is clearly from the D1-DHN sample, coherently with the results of Fig. 9, the maximum photogenerated current is significantly reduced over time for both kinds of samples, further indicating that a longer initial treatment is mandatory to achieve optimized results.

The effect of the wet/dry environment on the melanin/PSi samples is an excellent test lab for the understanding of the conducting properties of the melanin polymers. Since, as already indicated earlier, the dynamics of the changes in the polymer conductivities is slow and take several days to saturate, we resorted to a computational modelling of the mobility of water molecules within the polymer.

### Computational results

The computed density profile of the polymer along the direction perpendicular to the substrate showed a large increase with respect to the bulk value (larger than 100%) in the region of the polymer having a thickness of about 1.3 nm. For larger distances between 1.3 and 5 nm the density is generally larger than bulk, while for distances larger than 5 nm the overall density recovers the one of the bulk polymer^54^.

We investigated the effect of such a density variation along the direction perpendicular to the substrate on the water diffusivity inside the polymer matrix. To this aim we considered two different initial conditions by considering the diffusion of 100 TIP3P water molecules^73^ initially placed inside the polymer matrix in a region (i) named R1, between z > 0 (corresponding to the polymer/Si interface) and z < 1.3 nm and (ii) in a region named R2 corresponding to z > 2 and < 4 nm.

We estimated the water diffusion coefficient by performing a simulation as long as 4 × 10^5^ MD steps at T = 300 K during which we monitored the time dependence of the water mean square displacement (MSD) in both cases.

**Figure 11:**
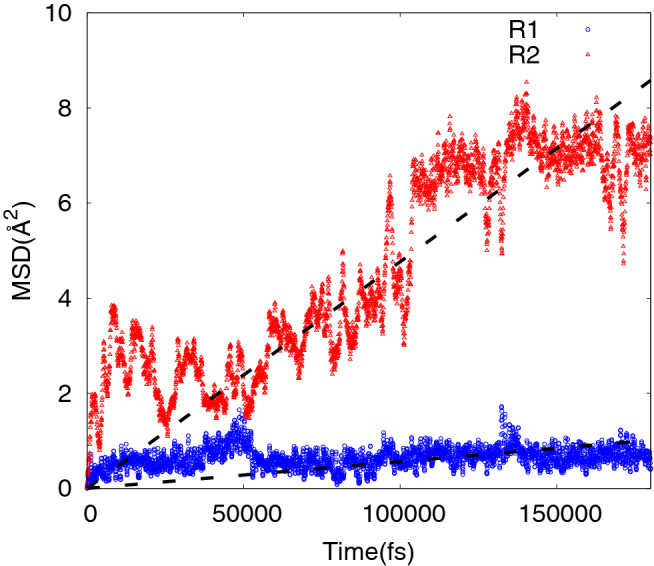
Estimated MSD versus time at T = 300 K for 100 TIP3P water molecules initially placed inside the polymer matrix in a region R1 between z > 0 and z < 1.3 nm (blue circles) and in a region R2 corresponding to z > 2 and < 4 nm (red diamonds).

Figure 11 shows the MSD vs time during the last 180 ps of the simulations where a linear dependence was observed in both cases. By considering the Einstein formula it is then possible to estimate the corresponding diffusion coefficients in both cases. In detail we obtain D(R1) = 9.4 × 10^–8^ cm^2^/s and D(R2) = 7.94 × 10^–7^ cm^2^/sec for regions R1 and R2 respectively.

In both cases the water diffusion coefficient is dramatically reduced (by more than 2 orders of magnitude) with respect to the water self-diffusion coefficient^81^ of 2.57 × 10^–5^ cm^2^/s. Moreover, we observe that the water diffusivity in the region close to the Si/eumelanin interface is strongly reduced (by more than one order of magnitude) with respect to the diffusivity in the polymer bulk region where the mass density is much lower with respect to the one at the interface.

The computational results show that the water mobility is a slow process and therefore support the experimental evidence of a wetting/drying process requiring several days to reach an equilibrium. In addition, they show that the water molecules mobility is significantly slower in the polymer portions where the density is increased, that is up to a distance of about 5 nm from the Si/polymer interface. This further support our interpretation of the results that the improved response of the hybrid system to the wet/dry cycling is only possible where the unaffected polymer volume is sufficiently large to allow for the “natural” polymer behavior.

## Conclusions

The combined experimental and computational study presented here gives several new insights from the point of view of the understanding the PSi/melanin hybrid behavior but also for the understanding of polymer properties alone. Those new insights are tightly related to the design of melanin/PSi organic/inorganic hybrid structures having potential applications in the renewable energy field, e.g. solar power collection.

First, we demonstrate that the polymer inserted within large (i.e. 40–60 nm in diameter) pores recover its bulk properties. This is explained by the fundamental role played in the overall hybrid system behavior by the densification of the polymer near the Si/polymer interface.

Second, we show that the initial treatment also is determinant for the final structure properties and lifetime. While the full explanation of this behavior requires further investigations, it is evident that, as before, a polymer structural modification has to play a major role, in particular to account for the dramatic difference in the dry vs. vacuum initial treatment.

Third, our results indicate that the overall polymer conductivity changes do not depend on a difference in the bulk properties but, more likely, on a difference in the polymer portion closer to the external surface, since the low mobility of water molecules suggests that these portions are the most affected by the environment changes. Since these areas are also those in touch with the electrical contacts of the structure, their influence on the measured properties is clearly larger.

Fourth, our experiments show that using PSi as a tool to understand the properties of a polymer inserted within its pores may be a powerful tool to access information that would be otherwise hinted by the geometry of the systems. As a matter of fact, in this study we were able to investigate experimentally the behavior of very thin polymer layers, whose thickness can be assumed equal to half of the average pore diameter. This means that by using PSi as a tool we gained previously inaccessible experimental information on polymer thickness in the 5–30 nm range.

Further investigations are therefore needed to fully explain the origin of the melanins sensitivity to the humidity level, and the understanding of the polymer behavior at the near-surface regions will play a major role, given the precious insights that they can give to the experimental determination of the polymer properties when structural modifications intervene. The use of porous structures both to play an active role in the final structure and give access to nanoscale information on the polymer properties, combined with an integrated experimental and computational information, gave unprecedented experimental access to the nanoscale behavior of the melanic polymers and therefore constitute a fundamental step to design efficient hybrid structures and devices for practical applications, e.g. energy conversion applications.

## Data Availability

All data needed to evaluate the conclusions in the paper are present in the paper. Additional data related to this paper may be requested from the authors.

## Abbreviations

AISSP: Ammonia-induced solid-state polymerization
DHI: 5,6-Dihydroxyindole
DHN: 1,8-Dihydroxynaphthalene
ENL: Electrochemical NanoLithography
EtOH: Ethanol
MSD: Mean square displacement
PANI: Polyaniline
PEDOTPSS: Poly(3,4-ethylenedioxythiophene) polystyrene sulfonate
PSi: Porous silicon
RESP: Restrained electrostatic potential
